# Quantifying the natural history of post-radical prostatectomy incontinence using objective pad test data

**DOI:** 10.1186/1471-2490-7-2

**Published:** 2007-02-05

**Authors:** Anna R Smither, Michael L Guralnick, Nancy B Davis, William A See

**Affiliations:** 1 Currently at Louisiana Urology, LLC, Baton Rouge, LA, USA; 2 Department of Urology, Medical College of Wisconsin, Milwaukee, WI, USA; 3 Division of Neoplastic Diseases & Related Disorders, Medical College of Wisconsin, Milwaukee, WI, USA

## Abstract

**Background:**

Urinary incontinence (UI) following radical prostatectomy is a well-recognized risk of the surgery. In most patients post-operative UI improves over time. To date, there is limited objective, quantitative data on the natural history of the resolution of post-prostatectomy UI. The purpose of this study was to define the natural history of post radical prostatectomy incontinence using an objective quantitative tool, the 1-hour standard pad test.

**Methods:**

203 consecutive patients underwent radical prostatectomy by a single surgeon between 03/98 & 08/03. A standardized 1-hour pad test was administered at subsequent postoperative clinic visits. The gram weight of urine loss was recorded and subdivided into four groups defined according to the grams of urine loss: minimal (<1 g), mild (>1, <10 g), moderate (10–50 g) and severe (>50 g). Patients were evaluated: at 2 weeks (catheter removal), 6 weeks, 18 weeks, 30 weeks, 42 weeks and 54 weeks. The data set was analyzed for average urine loss as well as grams of urine loss at each time point, the percentage of patients and the distribution of patients in each category.

**Results:**

Mean follow up was 118 weeks. The majority of patients experienced incontinence immediately after catheter removal at 2 weeks that gradually improved with time. While continued improvement was noted to 1 year, most patients who achieved continence did so by 18 weeks post-op.

**Conclusion:**

While the majority of patients experience mild to severe UI immediately following catheter removal, there is a rapid decrease in leaked weight during the first 18 weeks following RRP. Patients continue to improve out to 1 year with greater than 90% having minimal leakage by International Continence Society criteria.

## Background

Urinary incontinence is one of the most devastating and feared complications of radical retropubic prostatectomy (RRP). Incontinence following RRP has been reported to occur in 2.5–87% of patients [1]. The wide range is the result of a variety of factors including different definitions of incontinence and methods of assessment. For example, patient self reported incontinence appears to be greater than that reported by physicians [2]. However, it has also been shown that patients' estimates of incontinence severity are inaccurate particularly when compared with more objective measures of incontinence such as pad testing [3,4].

While the majority of patients experience incontinence immediately following RRP, in many this is transient, with a gradual improvement over time [5,6]. For those who fail to achieve adequate continence, treatments such as the artificial urinary sphincter and male sling are available which can significantly improve continence and quality of life. However, it is generally recommended that one wait at least 6–12 months before proceeding with these treatments because of the potential for spontaneous improvement [7]. Yet this condemns those patients who do not achieve continence to a prolonged period of incontinence with resultant reduction in quality of life. Ideally, one would like to be able to identify those patients who are not likely to achieve continence early so that one can intervene sooner. In order to do so, one needs to determine the natural history of incontinence after radical prostatectomy. The purpose of this study is to document the natural history of post-prostatectomy incontinence over time using an objective measure of incontinence assessment, the 1 hour pad test.

## Methods

The accumulated pad test data (obtained prospectively) was reviewed from the charts of 203 consecutive patients who underwent radical retropubic prostatectomy (RRP) by a single surgeon between 3/98 and 8/03. The Froedtert Hospital Institutional Review Board granted approval for the review. Patient demographics including age, pre-op PSA, and clinical stage were recorded. An attempt at anatomic nerve sparing radical prostatectomy using the Walsh technique was performed in all patients with the urethrovesical anastomosis performed in an intussuscepted fashion as described by See et al [8]. Patients were instructed in pelvic floor exercises (verbally and with hand-out) pre-operatively and again at the time of catheter removal, 2 weeks post-operatively. No additional interventions were made (e.g. biofeedback). All patients underwent standardized 1 hour pad tests as per the International Continence Society to assess post-operative incontinence (Appendix 1) [9,10]. These were performed at 2 weeks (time of catheter removal), 6 weeks, 18 weeks, 30 weeks, 42 weeks, and 54 weeks post-operatively. The gram weight of urine loss at each time point was recorded for each patient. Severity of incontinence was categorized according to the grams of urine loss as follows: minimal/continent (≤1 g), mild (1.1–9.9 g), moderate (10–49.9 g), severe (≥50 g) as per ICS recommendations. The percentage of patients in each category was calculated. Patients who achieved less than 1 g on a pad test and then refused to participate in subsequent evaluations were assumed to have the same degree of incontinence on those subsequent visits for purposes of analysis.

## Results

**Figure 1:**
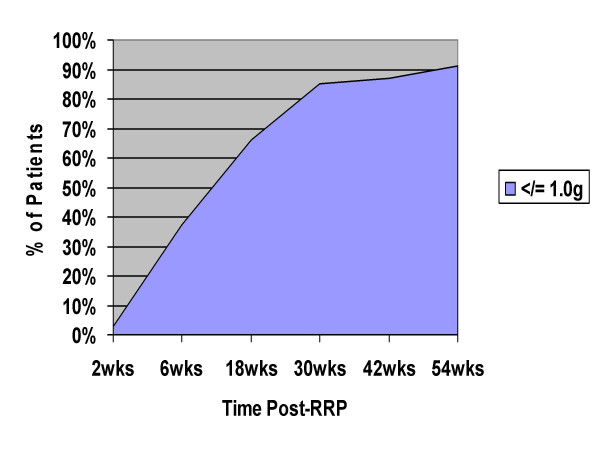
Continence Recovery Over Time.

Patient age ranged from 40–72 years (mean 58) and pre-op PSA ranged from 0.3–25.4 (mean 7.3). All had clinically localized prostate cancer: 0.4% T1a, 1.6% T1b, 66% T1c, 26% T2a, 6% T2b. Table 1 shows the mean pad weight (i.e. weight of urine loss) of the entire population over time. There was a rapid improvement in urinary control during the first 18 weeks post-RRP with a flattening of the recovery curve beyond that point as demonstrated in figure 1. Table 2 shows the percentage of patients in each severity of incontinence category at each time point. Again, the 18 week marker appears to be the time point after which the majority of patients have achieved urinary control such that at the 30 week assessment, 85% of patients fit in the minimal incontinence category (<1 g of leakage) and by 54 weeks, 91% of patients are in that category. Only 2 patients developed anastomotic contracture and both ended up with ≤1 g of leakage on final pad testing.

## Discussion

The causes of incontinence following radical prostatectomy include urethral sphincter damage, alteration in bladder function (e.g. detrusor overactivity) or a combination of both. However, most would agree, that sphincteric incompetence is likely the predominant factor [11]. Factors that have been associated with an increased risk for post-prostatectomy incontinence include: older age [5,12], advanced stage [12], and the presence of anastomotic stricture [5]. While most patients experience transient incontinence after catheter removal, many studies report an achievement of urinary continence in the majority of patients that can take up to 2 years [5,6], although in the majority this appears to occur within the first 6 months [13,14]. There is some evidence that nerve sparing can improve continence rates [15]. All our patients underwent an attempt at nerve sparing but we do not have the data regarding the number with attempted unilateral versus bilateral nerve sparing nor regarding the actual success of nerve sparing. Burkhard et al's data suggests there may not be significant difference in continence outcome between bilateral and unilateral nerve sparing (1.3% vs. 3.4% incontinence rates, respectively) but there was a significant difference when compared to non-nerve sparing (13.7% rate of incontinence) [15]. As such, our data needs to be considered in light of the nerve sparing approach and may not be generalizable to non-nerve sparing RRP.

Similar to other reports, our patients experienced a gradual improvement in their incontinence based on 1 hour pad testing such that by 16 weeks postoperatively, 2/3 of the patients had ≤1 g of leakage on their pad test consistent with being continent as per the ICS. By 30 weeks 85% of patients fit in this category and by 54 weeks, 91%. A study by Donnellan et al that is similar to ours in that 1 hour pad testing was performed, showed almost the identical results with 64% objectively dry at 3 months, 72% at 6 months and 84% at 1 year [13]. In our study, all patients were instructed on pelvic floor exercises pre-operatively as well as post-operatively and this has been shown by some to hasten continence recovery [16] although not by others [17]. We do not have data regarding the compliance with the exercises which is a limitation of the study and furthermore we do not have a comparison group that was not instructed. Filocam et al's study of pelvic floor exercises versus no intervention showed a similar trend in continence recovery as our study with the greatest improvement in incontinence occurring within the first 6 months of surgery [16]. In fact 74% of patients performing pelvic floor exercises were considered continent at 3 months while 30% of patients who did not perform the exercises were continent. Our continence rates fall somewhere in between these 2 points and perhaps reflect the fact that our patients did not have as comprehensive teaching in pelvic floor exercises as their patients. It is noteworthy that while pelvic floor exercises may have benefit with respect to continence recovery time, they do not appear to influence continence rates at 1 year or beyond [16].

We did not incorporate a patient estimate of incontinence severity or pad usage at home because the purpose of this study was to specifically use an objective measure of incontinence with the 1 hr pad test. Quantitative measurement of urine leakage was introduced by James et al [18] and ultimately developed into the 1 hour pad test by Bates et al [10]. This was subsequently endorsed by the International Continence Society in 1988 [9]. Studies have been inconclusive as to the patient's ability to estimate incontinence severity with some claiming a good correlation between pad usage and degree of incontinence [6], while others showed a poor correlation [3,4]. There are a number of variables that factor into this equation including: variable pad size, degree of wetness, and patient tolerability of wetness resulting in more/less frequent pad changes. Furthermore, some patients may prefer not to use pads at all yet still experience some incontinence for which they change their underwear/clothes; they would be categorized as continent on the basis of lack of pad use if that is the definition of continence, which it often is in studies. But of course they are not technically continent. What is clear is that continence rates vary significantly based on the use of different definitions of incontinence and the method of assessment (e.g. patient reported versus physician reported) [2,19]. Some have argued that with respect to quality of life, simply asking the patient about incontinence and the subjective severity may be as good as or better than using a pad test to quantify incontinence [20]. However, we would argue that patients experience incontinence differently and what might be considered mild, non-problematic incontinence by one patient may not be the same to another patient. In one study, while 63% of patients continued to be incontinent 6 months postoperatively with a median leakage of 46 mL on 24 hour pad testing, 71% of patients found their incontinence to be of no or only a minor problem on quality of life assessment [3]. The authors argue, therefore, that the degree of bother may be the best indicator of severity of incontinence. However, we would argue that the attitude toward incontinence is different for different patients. Hence an objective measure of incontinence severity is needed. This is evidenced by the fact that some patients insist on frequent pad changes for minimal incontinence while others may not even wear a pad despite significant incontinence. Thus simply relying on patient reported pad usage can potentially over- or underestimate the actual severity of the incontinence. At least with pad weighing, one gets an absolute value as to the degree of incontinence. However, we do agree that before any aggressive intervention for incontinence, the degree of bother must be taken into account; there would be no indication for surgical intervention if the patient is not bothered by his incontinence. Our study, therefore, is limited by this lack of subjective assessment of the degree of bother but nevertheless does provide important objective data.

Whether a 1-hour pad test is as reliable as a more prolonged pad test such as a 24 or 48 hour test is still a matter of debate, and while some have argued that a 24 hour test is more useful [21,22], the 1-hour pad test does require less patient compliance because it can be done at a clinic visit and does not burden the patient with home instructions that can easily be misinterpreted or forgotten. It has been demonstrated that the longer the pad test is kept the less compliant are patients [23]. Our pad test was done following the consumption of a standard volume of fluid but bladder volumes were not standardized. It is known that the reliability of the 1 hour pad test can be increased when bladder volumes are standardized [24] and therefore this is another limitation of our study. The limit of 1 g of leakage that we used as an indicator of continence is based on the results in women since to our knowledge there is no data on this in men. However, Moore et al, using the 24 hour pad test in men who considered themselves continent prior to RRP, found an upper limit of 8 g/24 hr as being a reliable indicator of continence and this is generally considered the upper limit for continence in women on a 24 hour pad test as well [25]. As such we believe that the 1 g threshold for the 1 hr pad test is reasonable for men.

Our ultimate goal is to be able to use continence recovery time nomogram to help counsel patients as to the likelihood of achieving continence post-RRP. A prospective evaluation is underway looking at the predictive value of this nomogram. If it is determined at the 3 or 6 month time point that the likelihood of achieving continence is extremely low, the patient may opt for more aggressive treatment (e.g. artificial urinary sphincter, AUS) sooner rather than later which might allow for improved quality of life sooner rather than later. Currently, it is recommended that patients wait at least 6–12 months from their RRP before undergoing AUS placement [7]. However, if our nomogram is accurate in its ability to predict the achievement/failure to achieve continence, then patients may make decisions regarding the timing of definitive intervention based upon the probability of recovery. This could translate into an improvement in quality of life sooner. Because surgical skill and technique are clearly important variables with respect to outcome [26,27], it is probably best that surgeons review their own experience to perhaps devise a nomogram as we have done to be better able to counsel their patients regarding their incontinence.

## Conclusion

Using an objective measure of incontinence, we have tracked continence recovery following RRP and we confirm the results of others that the majority of patients experience incontinence after radical prostatectomy that gradually improves with time. By 18–30 weeks post-operative, most patients experience minimal to no incontinence. A prospective study is underway to evaluate the ability of our nomogram to predict continence recovery. If the predictive ability is reliable, we may be better able to counsel patients as to the likelihood of continence recovery which may allow for earlier intervention in those patients who are unlikely to achieve satisfactory continence.

## Competing interests

The author(s) declare that they have no competing interests.

## Authors' contributions

AS participated in the design of the study, data acquisition and analysis and helped to draft the manuscript

MG participated in the design of the study, data analysis and helped to draft the manuscript

ND helped to draft and edit the manuscript

WS conceived of the study, and participated in its design and coordination and helped to draft the manuscript.

All authors read and approved the final manuscript.

## Appendix 1

One-hour pad test

1) test is started without the patient voiding

2) preweighed pad is put on and 1-hour test period begins

3) 0–15 minute: subject drinks 500 mL of water and sits or rests

4) 15–45 minutes: subject walks, including stair climbing equivalent to one flight up and down

5) 45–60 minutes: subject performs the following activities:

a. Standing up from sitting, 10 times

b. Coughing vigorously, 10 times

c. Running on the spot, 1 minute

d. Bending back to pick up small object off the floor, 5 times

e. Washing hands in running water, 1 minute

6) At the end of the 1-hour test the pad is removed and weighed

## Pre-publication history

The pre-publication history for this paper can be accessed here:



## Figures and Tables

**Table 1:** Mean Pad Weight on 1 hr Pad Test at Each Time Point

Time	Mean Pad Wt. in Grams (range, SD)
2 weeks	36.7 (0–241.8, 40.5)
6 weeks	21.9 (0–226, 39.5)
18 weeks	5.6 (0–76, 14.2)
30 weeks	2.6 (0–80.4, 10.6)
42 weeks	2.8 (0–104.5, 14.1)
54 weeks	1.7 (0–71.1, 8.9)

**Table 2:** Percentage of Patients Within Each Incontinence Category at Different Time Points of Assessment (based on pad weights)

	Incontinence Severity			
	
Time	Minimal (≤1.0 g)	Mild (1.1–9.9 g)	Moderate (10.0–49.9 g)	Severe (≥50.0 g)
2 weeks	3%	23%	50%	24%
6 weeks	37%	23%	26%	14%
18 weeks	66%	22%	8%	4%
30 weeks	85%	9%	4%	2%
42 weeks	87%	8%	3%	2%
54 weeks	91%	5%	3%	1%
